# Pharmacological and Computational Insights Into the Analgesic, Antipyretic, and Antidiarrheal Potential of *Mallotus paniculatus* Acetone Extract

**DOI:** 10.1155/tswj/9813151

**Published:** 2026-03-03

**Authors:** Md. Jahirul Islam Mamun, Md. Abdul Alim, Sadia Tamanna Tamim, Md. Iqbal Hossen, Sajib Chandro Das, Tanbirul Azim Maharaj, Dipta Debnath, Sajeda Akter, S. M. Moazzem Hossen

**Affiliations:** ^1^ Department of Pharmacy, Faculty of Biological Sciences, University of Chittagong, Chittagong, Bangladesh, cu.ac.bd; ^2^ Department of Pharmacy, BGC Trust University Bangladesh, Chattogram, Bangladesh, bgctub-edu.com; ^3^ Department of Pharmacy, International Islamic University Chittagong, Chittagong, Bangladesh, iiuc.ac.bd

**Keywords:** analgesic, antidiarrheal, antipyretic, docking, *Mallotus paniculatus*

## Abstract

*Mallotus paniculatus* (Lam.), a member of the Euphorbiaceae Juss family, is a little tree or shrub that has long been used in folk medicine to cure ailments like fever, wound healing, and postpartum recuperation. In this study, Swiss albino mice were used to evaluate the analgesic, antipyretic, and antidiarrheal properties of the acetone extract of *M. paniculatus* (AMP). Standard screening techniques were used to identify the phytochemical ingredients. Three models were used to evaluate analgesic activity: the tail immersion test, formalin‐induced paw licking, and acetic acid–induced writhing. Antipyretic activity was assessed using a fever model induced by brewer′s yeast. At the same time, antidiarrheal effects were evaluated by castor oil–induced diarrhea, and gastrointestinal motility was studied using a charcoal meal marker. Furthermore, in silico analyses—such as molecular docking, ADME profiling, toxicity prediction, and PASS analysis—were conducted using online tools. The results indicated that AMP at a lower dose (200 mg/kg) produced significant analgesic effects across all pain models compared with the control group. In the antipyretic evaluation, AMP administered at 400 mg/kg illustrated the most pronounced reduction in body temperature after 4 h, which was statistically significant (*p* < 0.01). The same higher dose (400 mg/kg) also significantly reduced diarrheal episodes and slowed gastrointestinal motility in both the castor oil–induced diarrhea and charcoal meal transit tests. Molecular docking analysis further corroborated these pharmacological effects, revealing that AMP compounds exhibited strong binding affinity toward key target receptors associated with pain, inflammation, and gastrointestinal activity. Collectively, these results indicate that AMP has promising potential as a natural multitarget therapeutic agent for the treatment of pain, fever, and diarrhea.

## 1. Introduction

Diarrheal diseases continue to pose a significant global health challenge, significantly contributing to morbidity and mortality, particularly in developing countries, where children are most severely affected [1]. Several microorganisms are key causal factors of diarrheal diseases in humans, including *Shigella flexneri*, *Staphylococcus aureus*, *Escherichia coli, Salmonella typhi*, and *Candida albicans* [2, 3]. Although various conventional treatment options are available for managing diarrheal conditions, a large number of individuals in developing countries continue to rely essentially on classic herbal or botanical therapies for relief. In response, the World Health Organization (WHO) has endorsed research into traditional medicine systems to identify practical, accessible, and affordable treatments for diarrheal disorders, particularly in resource‐limited settings [4]. Diarrhea is commonly caused by gastrointestinal infections resulting from various bacteria, viruses, and parasites. It can be transmitted through contaminated food, drinking water, and unsanitary environmental conditions. In addition to other underlying pathological conditions, the pathophysiology of diarrhea often involves disturbances in water and electrolyte transport, primarily driven by four key mechanisms: increased luminal osmolarity, enhanced electrolyte secretion, reduced electrolyte absorption, and accelerated intestinal motility, all of which contribute to shortened transit time and impaired fluid reabsorption in the gut [5]. Despite efforts by international organizations to control and reduce the burden of diarrhea, it remains a highly prevalent condition worldwide, particularly in low‐ and intermediate‐income economies [6]. Although certain antibiotics are used as antidiarrheal drugs, they can occasionally cause adverse side effects and cause bacteria to develop resistance to them [7].

The body′s inflammatory processes might cause fever or pyrexia as a side effect [8]. The amplified production of prostaglandins functions as the key mechanism that initiates pain, inflammatory responses, and fever development. As a result, the majority of anti‐inflammatory compounds are expected to exhibit both pain‐relieving and temperature‐lowering effects through their ability to prevent or limit prostaglandin overproduction [9]. Given the adverse reactions caused by NSAIDs and opioid analgesics, there is considerable interest in developing alternative drug classes with reduced or absent side effects. Consequently, present research directions have gravitated toward traditional plant medicines due to their economical nature, easy availability, and diminished risk of harmful effects [10]. A typical computational approach in drug discovery, docking methodology optimizes large drugs and finds new active molecules through virtual screening, in addition to small molecule experimental binding modes and affinities [11]. In molecular docking, search techniques are employed to determine the optimal ligand postures and explore the potential energy surface [12]. To predict the complexation affinities and binding modalities of biological molecules with a specific target receptor, a structure‐based approach utilizing ligand–receptor molecular docking is typically employed [12]. Medicinal herbs are widely used worldwide to treat a range of chronic illnesses, including mild fevers and life‐threatening conditions [13]. In Bangladesh′s cities, they are also a notable source of medication and play a crucial part in the treatment of several illnesses [14]. Scientifically solid experimental data are the primary source of support for the traditional folkloric use of medicinal herbs. Numerous physiologically active secondary metabolites with a high potential for medical use are found in the Euphorbiaceae family, also known as the spurge family. Alkaloids and amides are among the several natural chemicals found in many paniculata species [15]. Approximately one‐quarter of the drugs available today are derived from plants, and their pharmacological and biochemical properties provide strong evidence for their medicinal use [16].

*Mallotus paniculatus* (Lam.), a species of the family Euphorbiaceae Juss., is a shrub or small tree that was initially described by Lamarck in 1865 [17]. It is primarily found in regions such as Bangladesh, India, Malaysia, China, New Guinea, and Australia [18–20]. In rural areas, the *M. paniculatus* (commonly known as balik angin) plant has been traditionally used for a long time to treat various ailments, including fever, wound healing, and postpartum recovery [18]. The plant is utilized in the Guangxi region to address a range of medical conditions, including otitis media and diarrhea [17]. The plant extract has been found to exhibit antibacterial, anticancer, antioxidant, and antiradical properties. Previous studies on the chemical constituents of *M. paniculatus* have led to the identification of several bioactive compounds, including cardenolides, triterpenoids, steroids, flavonoids, and unsaturated fatty acids [20]. The present study was designed to assess the bioactive phytochemicals and the analgesic, antipyretic, and antidiarrheal properties of the acetone extract of *M. paniculatus* (AMP) using in vitro, in vivo, and in silico techniques, as no prior studies have explored these effects.

## 2. Materials and Methods

### 2.1. Drugs and Chemicals

In this study, analytical‐grade materials were utilized. Loperamide, diclofenac sodium, morphine sulfate, sodium pentobarbital, and Tween 80 were obtained from SRL, India, whereas the other chemicals were sourced from the University of Chittagong′s pharmacy department.

### 2.2. Plant Collection and Identification

Dried *M. paniculatus* leaves were collected in June 2024 from the rural region of BGC Trust University, Chattogram, Bangladesh. Professor Dr. Shaikh Bokhtear Uddin of the University of Chittagong in Bangladesh confirmed the taxonomy of the samples. After being assigned as Accession Number 3735, an herbarium specimen was housed at the Herbarium Centre of the University of Chittagong.

### 2.3. Extraction Methods

The assembled leaves were cleaned and shade‐dried at approximately 25°C for 4 weeks before undergoing further processing. Two kilograms of dried *M. paniculatus* leaves were crushed in a blender after being ground in a mortar. Two litres of 95% acetone were used to macerate 500 g of material for 3 days. The crude residue was subjected to two additional iterations of this macerated process. The extract was filtered using cotton and Whatman paper filters. A rotary evaporator (RE200, Bibby Sterling, United Kingdom) was used to dry the filtrate. AMP leaves was kept in a glass vial at 4°C before the test [21].

### 2.4. Phytochemical Screening

The presence of various chemical groups, including alkaloids, glycosides, steroids, carbohydrates, tannins, flavonoids, and saponins, was qualitatively examined in all of the AMP [22–24].

### 2.5. Experimental Animal

Swiss albino mice weighing between 22 and 30 g were acquired from the animal research department of Rajshahi University in Bangladesh. They were kept in polycarbonate cages with a 12‐h light/dark cycle and standard conditions (25^°^C ± 2^°^C, 55%–60% humidity) [25]. The animals were provided with unlimited access to food and water throughout the study. The animal experiment was approved by the University of Chittagong′s departmental ethical review committee with Clearance Number ERC/CUDP/2024/06 and was conducted in strict compliance with the ARRIVE guidelines to uphold ethical standards, methodological rigor, and reporting transparency.

### 2.6. Experimental Design

For the study, male and female Swiss albino mice were used in the test, control, and standard sets. Each experimental group consisted of five mice, and the control group received 10 mL/kg of a 1% Tween 80 solution in water. The test groups were administered oral AMP at 200 and 400 mg/kg of total body weight, respectively, using the oral gavage technique. For the analgesic tests, diclofenac sodium (10 mL/kg) was used as a standard for the acetic acid–induced writhing and formalin‐induced paw licking test, as well as morphine sulfate (10 mL/kg) used as a standard for the tail immersion test. Paracetamol (100 mg/kg) was used as the standard for the antipyretic test, and loperamide (1 mg/kg) was the standard medicine for the antidiarrheal tests.

### 2.7. Animal Euthanasia

All animal procedures were approved by the University of Chittagong′s IACUC and adhered to national and institutional guidelines. Euthanasia was performed via intraperitoneal injection of freshly prepared sodium pentobarbital (200 mg/kg). Animals were gently handled, monitored for anesthetic depth (loss of righting, pedal withdrawal, and corneal reflexes), and confirmed dead upon irreversible cessation of respiration and heartbeat. A secondary method (bilateral thoracotomy) was applied per AVMA guidelines to ensure humane death. All personnel were trained and competent, and carcasses were disposed of following institutional biosafety protocols.

### 2.8. Acute Oral Toxicity

An acute oral toxicity study was conducted in accordance with the Organisation for Economic Co‐operation and Development (OECD) Guideline for the Testing of Chemicals, Test No. 423 [26]. The study involved five female rodents (typically fasted overnight), which were administered a single administration by mouth of AMP at 1000, 2000, or 4000 mg/kg body weight via oral gavage. Following administration, food was withheld for 3–4 h to minimize gastrointestinal interactions. Animals were closely monitored during the first 30 min postdosing and then observed periodically over the next 24 h for any signs of toxicity. Observations were continued daily for a total of 3 days to detect any delayed adverse effects. Parameters assessed included changes in skin, fur, eyes, mucous membranes, respiratory and circulatory rates, as well as autonomic and central nervous system (CNS) activity. No mortality or significant behavioral or physiological alterations were observed throughout the study period. The median lethal dose (LD_50_) was determined to be greater than 2000 mg/kg (LD_50_ > 2000 mg/kg), indicating that AMP is practically nontoxic under acute exposure conditions [27]. This result was used to establish safe dose levels for subsequent pharmacological studies, with the therapeutic doses selected well below the toxic threshold.

### 2.9. Analgesic Activity

#### 2.9.1. Acetic Acid–Induced Writhing Test

A common technique for assessing *M. paniculatus*′s analgesic efficacy was the acetic acid–induced writhing method [28, 29]. Two doses of the test extracts (200 and 400 mg/kg body weight), control (10 mL/kg body weight), and diclofenac sodium (10 mg/kg body weight) were administered orally 40 min before the intraperitoneal injection of 0.7% acetic acid, following an overnight fast. The total number of writhes for each animal was recorded over 15 min, starting 5 min after the acetic acid was administered.

%Inhibition=Wc−Ws Wc×100

Here, *W* *c* means the number of writhing in the control group, and *W* *s* means the number of writhing in the sample group.

#### 2.9.2. Formalin‐Induced Licking Test

The prior work provided a detailed description of the experimental apparatus used in this investigation [30]. Four groups of mice were created: Group 1 received 1% Tween 80 (1 mL/kg) intraperitoneally (i.p.), Group 2 received standard treatment (diclofenac sodium, 10 mg/kg), and Groups 3 and 4 received treatment with AMP at 200 and 400 mg/kg body weight, respectively. Thirty minutes following this treatment, 50 *μ*L of a freshly prepared 0.6% formalin solution was subcutaneously injected into the plantar region of each mouse′s left hind paw. All of the mice were observed for an hour. Paw biting and licking duration (s) is a measure of discomfort response. The antinociceptive effect was assessed in two phases. The first 5 min following the formalin injection were noted as the early phase (Phase 1), and the last 30 minutes as the late phase (Phase 2) [31]. The following formula was used to get the inhibition (%):

Inhibition %of licking=total number of licking  control−test groupcontrolx 100

#### 2.9.3. Tail Immersion Test

The tail immersion method, which was devised and described by Gawel et al. [32] was used to evaluate the central analgesic property. This approach is predicated on the finding that medications with morphine‐like effects can prolong the mice′s tail′s abandonment from warm water. One to two centimeters of the rodents′ tail was immersed in hot water that was kept at 54^°^C ± 0.5^°^C after the animal had been treated with diclofenac sodium or extracts. The latency duration of the tail‐withdrawal reaction was measured at 30, 60, 90, and 120 min following the administration of the medication and extracts. To prevent harm to the tail tissue, the latency period was limited to 20 s [33].

### 2.10. Antipyretic Activity

#### 2.10.1. Yeast‐Induced Pyrexia Method

The antipyretic effect of the AMP was assessed using the brewer′s yeast–induced pyrexia technique on mouse models by injecting a yeast suspension subcutaneously at a dose of 10 mL/kg body weight [34]. Even though the animal models in this study had unrestricted access to water, they were not allowed to eat anything the night before the experiment. An Ellab thermometer was used to record the initial rectal temperatures [35]. Mice were only selected for the assessment of antipyretic properties if, following an 18 h subcutaneous injection of yeast solution, their rectal temperatures rose by 0.3°C–0.5°C. The control group received distilled water (10 mL/kg), whereas *M. paniculatus* extracts were administered orally at two different doses (200 and 400 mg/kg body weight) in addition to paracetamol (100 mg/kg body weight) as the conventional medication. Lastly, the tracking of rectal temperature continued for 3 h at 1‐h intervals [36].

### 2.11. Antidiarrheal Activity

#### 2.11.1. Castor Oil–Induced Diarrhea

The experiment began with an 18‐h fast for both male and female mice. As stated in Section 2.6, the dosage for the mice that were randomly separated was administered. The mice were placed in individual cages with blotting paper on the floor after an hour, and they were given castor oil (0.5 mL) orally by gavage. Every hour for 4 h, the quantity of dry and wet feces was recorded. They changed the blotting paper at the beginning of each hour. The following equation was utilized to determine the percentage of antidiarrheal activity inhibition (%) [37].

%Inhibition=A−BA×100

where A = mean number of diarrheal feces of the control group and B = mean number of diarrheal feces of the treated group.

#### 2.11.2. Gastrointestinal Motility Test

The charcoal transit test was conducted following the method described by Salawu et al. [38]. The mice of either sex were fasted for 12 h before the experiment, with access to water ad libitum. The animals were randomly divided into five groups, each consisting of three rats. Diarrhea was induced in all animals by oral administration of 1 mL of castor oil. At predetermined time intervals, an activated charcoal suspension was administered via oral gavage. One hour after treatment, each rat received 0.2 mL of a charcoal meal (10% activated charcoal in 5% gum acacia) orally. Thirty minutes after administration of the charcoal meal, the animals were euthanized. The small intestine was carefully excised from the pyloric sphincter to the ileo–caecal junction, freed from the mesentery to prevent stretching, and placed on a flat surface. The total length of the small intestine and the distance travelled by the charcoal bolus were measured in centimeters. Percent inhibition (PI) of gastrointestinal motility was calculated for each group using the following formula [39].

%Inhibition=mean control group−mean treated groupmean control group×100

### 2.12. In Silico Study

#### 2.12.1. Software Tools

The Protein Data Bank (PDB), Drug Banking, Swiss PDB Viewer, AutoDock Vina, MGL Instruments, PubChem, and Discovery Studio Visualizer 2021 (BIOVIA) were among the resources and technologies used in the analysis [40, 41].

#### 2.12.2. Selection of Ligands

The structures of AMP compounds (Supporting Information (available here)) were obtained from the PubChem database. In this study, phytochemicals produced from AMP were molecularly docked with the corresponding reference medications. To find the most hits, the ligands were virtually screened using PyRx [42].

#### 2.12.3. Validation of the Ligands

The selection of these substances as potential therapeutics is greatly influenced by their molecular and physical characteristics as well as pharmacokinetic parameters such as ADME/T (absorption, distribution, metabolism, excretion, and toxicity). Using the pKCSM online tool (http://biosig.unimelb.edu.au/pkcsm/), the potential of the listed compounds as ligands against therapeutic targets was verified on September 11, 2024 [43, 44]. The compounds were then assessed for drug potential using the Swiss ADME web server and Lipinski′s rule of five [45, 46].

#### 2.12.4. Protein Preparation

For the analgesic, antidiarrheal, and antipyretic test, the crystal structures of the target proteins—cyclooxygenase‐2 (COX‐2) (PDB: 6COX), muscarinic acetylcholine receptor M_3_ (PDB: 5ZHP), and microsomal prostaglandin E synthase‐1 (mPGES‐1) (PDB: 4YK5) were collected from the RCSB protein data bank. We utilized information previously published by Kurumbail et al. to identify the enzyme′s active location [47]. The necessary cleaning and preparation steps were performed using Swiss‐PdbViewer (v4.1) and the BIOVIA Discovery Studio 4.5 Client, which involved removing cofactors, heteroatoms, and water, respectively. After hydrogen atoms were added to the target protein′s structure, it was reduced using the PyRx virtual screening tool and a force field of MMFF94. The target protein was stored in pdb format to aid in docking research [48].

#### 2.12.5. Molecular Docking

PyRx 0.3 (http://pyrx.scripps.edu) (accessed September 11, 2024) and AutoDock, Version 4.2, were used for the docking analyses [49]. PyMOL was used to analyze the docking data. The type of contact (such as a hydrogen bond, *π*–*π*, or cation–*π* interactions) and its function in ligand binding can be identified thanks to these technologies. Additional details about the interaction between ligands and receptors were gathered using PyMOL (Protein–ligand docking: Current state and future challenges) [50, 51].

### 2.13. Pass Prediction

The PASS online tools were used to review the pass prediction to determine the possible biological effects of the chosen chemicals (http://www.pharmaexpert.ru/passonline/predict.php). The values of *p* *i* and *P* *a* varied between 0.000 and 1.000 [52]. Whenever the compounds′ *P* *a* values exceed their *P* *i* values, they are thought to have biological potential. Comparatively, *P* *a* > 0.7 indicates high pharmacological activity, *P* *a* < 0.5 indicates low pharmaceutical activity, and 0.5 < *P* *a* < 0.7 indicates moderate therapeutic potential [53, 54].

### 2.14. Statistical Analysis

The mean ± SEM (standard error of the mean) was used to describe the values. Statistical analysis was performed using one‐way ANOVA followed by Dunnett′s post hoc test. ∗ *p* <0.05, ∗∗ *p* <0.01, and ∗∗∗ *p* <0.001 revealed statistical significance when compared with the control group. For data analysis and graph drawing, GraphPad Prism 8.0.1 and Microsoft Excel 2024 were used. Five mice per group were used in the in vivo trial, whereas triplicate measurements were used in the in vitro study.

## 3. Results

### 3.1. Phytochemical Screening

A variety of phytochemicals were detected in AMP upon phytochemical screening, as shown in Table 1.

**Table 1:** Phytochemical analysis of the acetone extract of *M. paniculatus* leaves.

**Phytochemical constituents**	**Specific test**	**Inference**
Alkaloids	Mayer′s test	+
	Hager test	−
	Wagner test	−
	Dragendorff test	+
Carbohydrates	Benedict′s test	+
	Fehling′s test	−
Flavonoids	Alkaline reagent test	+
Phenols	Ferric chloride test	+
Saponin	Foam test	++
Tannins	Gelatin test	−
Glycoside	Liebermann′s test	−
Terpenoids	Chloroform test	+

*Note:* Presence (+); absence (−).

### 3.2. Acute Oral Toxicity

During the acute toxicity investigation, none of the tested doses showed any symptoms of toxicity or death. Throughout the monitoring time, the animals continued to consume food and water normally, and there were no discernible changes in their behavior, body weight, or general health, suggesting that there were no negative impacts. According to OECD rules, AMP is practically nontoxic, and its safety after oral administration has been confirmed by the finding that its LD_50_ is larger than 4000 mg/kg. Doses of 200 and 400 mg/kg were selected for further pharmacological testing based on this safety profile, ensuring efficient evaluation with a large safety margin.

### 3.3. Analgesic Activity

#### 3.3.1. Acetic Acid–Induced Writhing Test

**Figure 1:**
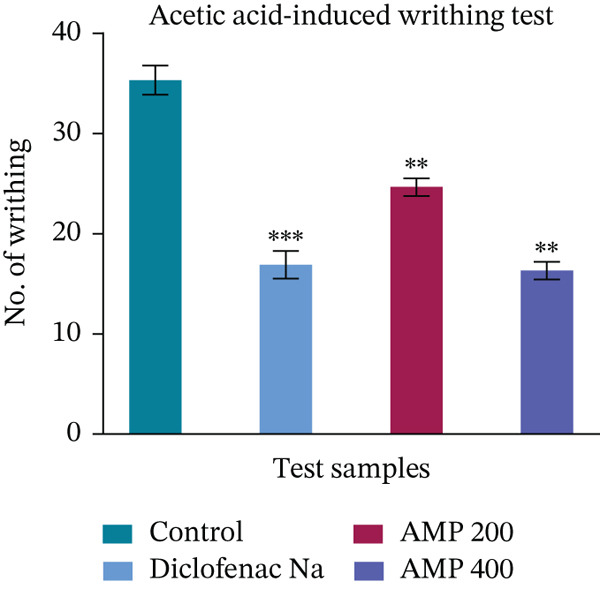
Evaluation of the analgesic activity of acetone extract of *M. paniculatus* (AMP) on the acetic acid‐induced writhing test.

In the acetic acid–induced writhing test, which evaluates peripheral analgesic activity by counting the number of abdominal constrictions (writhes) in mice, the control group displayed a high writhing count, averaging 35.33 ± 1.45 (Figure 1). This represents the normal pain response without any treatment. The analgesic efficacy of the test extract AMP varied with dosage. The number of writhes decreased to 24.67 ± 0.88 at a dose of 200 mg/kg, suggesting a 30.17% inhibition, which is less effective than diclofenac and a moderate decrease when compared with the control. Interestingly, increasing the dose to 400 mg/kg produced a much stronger effect, with writhes reduced to 16.33 ± 0.88 and a 53.77% inhibition, slightly surpassing the standard drug (Table 2).

**Table 2:** Effects of acetone extract of *M. paniculatus* (AMP) on acetic acid–induced writhing test.

**Test samples**	**No. of writhing**	**% of inhibition**
Control	35.33 ± 1.45	—
Diclofenac Na	16.9 ± 1.37∗∗∗	52.16
AMP 200	24.67 ± 0.88∗∗	30.17
AMP 400	16.33 ± 0.88∗∗	53.77

*Note:* Data are expressed as mean ± SEM (*n* = 5). Statistical analysis was performed using one‐way ANOVA followed by Dunnett′s post hoc test. AMP leaf acetone extract (AMP).

Abbreviation: AMP, acetone extract of *M. paniculatus*.

∗*p* < 0.05, ∗∗*p* < 0.01, ∗∗∗*p* < 0.001 indicate significant differences compared with the control group.

#### 3.3.2. Formalin‐Induced Paw Licking Test

In the formalin‐induced paw licking test, the extract AMP showed significant analgesic effects at 200 mg/kg, reducing licking time to 13 ± 1.15 s (56.18% inhibition) in the early phase and 8.67 ± 1.20 s (69.4% inhibition) in the late phase—exceeding diclofenac′s effect and surprisingly, increasing the dose to 400 mg/kg reduced efficacy, with only 18 ± 1.16 s (39.33%) and 14 ± 1.15 s (50.58%) in the respective phases. This paradoxical decrease suggests a nonlinear dose–response, where higher doses may saturate or modulate target pathways less effectively. The strong late‐phase inhibition indicates potent anti‐inflammatory or central analgesic activity at the lower dose (Table 3).

**Table 3:** Analgesic activity of AMP in the formalin‐induced paw licking test.

**Test samples**	**0**–**5 min (early phase)**		**20**–**30 min (late phase)**	
	**Paw licking time(s)**	**% of inhibition**	**Paw licking time(s)**	**% of inhibition**
Control	29.67 ± 1.20	—	28.33 ± 0.88	—
Diclofenac Na	11.8 ± 1.11∗∗∗	60.23	10.4 ± 0.75∗∗∗	63.29
AMP 200	13 ± 1.15∗∗∗	56.18	8.67 ± 1.20∗∗∗	69.4
AMP 400	18 ± 1.16∗∗∗	39.33	14 ± 1.15∗∗	50.58

*Note:* Data are expressed as mean ± SEM (*n* = 5). Statistical analysis was performed using one‐way ANOVA followed by Dunnett′s post hoc test.

Abbreviation: AMP, acetone extract of *M. paniculatus*.

∗*p* < 0.05, ∗∗*p* < 0.01, and ∗∗∗*p* < 0.001 indicate significant differences compared with the control group.

#### 3.3.3. Tail Immersion Test

**Figure 2:**
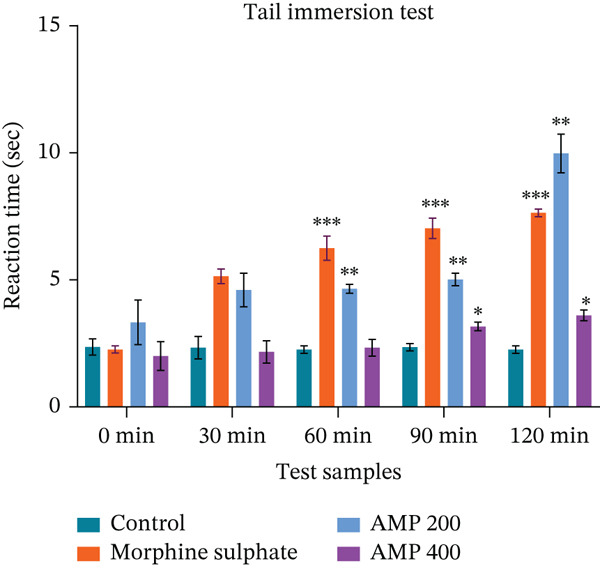
Analgesic activity of acetone extract of *M. paniculatus* (AMP) on the tail immersion test.

The tail immersion test assessed analgesic activity by measuring reaction time in hot water. The AMP 200 mg/kg group showed a delayed but significant increase in latency, peaking at 9.98 ± 0.76 s at 120 min, indicating potent central analgesia (Figure 2). In contrast, the 400 mg/kg dose produced only a modest rise, reaching 3.6 ± 0.21 s (*p* < 0.05 at 90 and 120 min). Despite dose escalation, the analgesic effect decreased, suggesting a nonlinear or biphasic response. These results indicate that the lower dose (200 mg/kg) elicits a more substantial, delayed analgesic effect, whereas the higher dose is less effective.

### 3.4. Antipyretic Activity

#### 3.4.1. Effect of AMP on Yeast‐Induced Pyretic Test

After an 18‐h subcutaneous administration of brewer′s yeast suspension, rectal temperature showed a significant rise. Mice treated with AMP (200 and 400 mg/kg) and paracetamol (100 mg/kg, i.p.) exhibited a significant (*p* < 0.05, 0.01, 0.001), dose‐ and time‐dependent reduction in fever compared with the control group. AMP at a dose of 400 mg/kg showed the highest inhibition of pyrexia after 3 h, with an effect comparable with that of the standard treatment, paracetamol (Table 4).

**Table 4:** AMP and control samples were shown to be protected from hyperpyrexia by brewer′s yeast in a mouse model.

**Treatment**	**Normal rectal temperature (°F)**	**Rectal temperature (°F) after yeast administration**	**Rectal temperature (°F) after drug administration**			
			**1** h	**2 h**	**3 h**	**4 h**
Control	97.33 ± 0.32	104.25 ± 0.23	104.05 ± 0.08	103.59 ± 0.24	102.31 ± 0.18	101.73 ± 0.57
Paracetamol	96.03 ± 0.30	103.85 ± 0.38	101.18 ± 0.10∗∗∗	99.26 ± 0.43∗∗∗	97.61 ± 0.51∗∗∗	96.49 ± 0.39∗∗∗
AMP 200	96.21 ± 0.45	104.15 ± 0.58	103.76 ± 0.16	102.01 ± 0.62∗	100.02 ± 0.71∗	99.30 ± 1.16
AMP 400	97.78 ± 0.85	103.93 ± 0.88	103.60 ± 0.24	101.54 ± 0.46∗∗	99.28 ± 0.66∗∗	98.14 ± 0.86∗

*Note:* Data are expressed as mean ± SEM (*n* = 5). Statistical analysis was performed using one‐way ANOVA followed by Dunnett′s post hoc test.

Abbreviation: AMP, acetone extract of *M. paniculatus*.

∗*p* < 0.05, ∗∗*p* < 0.01, and ∗∗∗*p* < 0.001 indicate significant differences compared with the control group.

### 3.5. Antidiarrheal Activity

#### 3.5.1. Castor Oil–Induced Diarrhea

**Figure 3:**
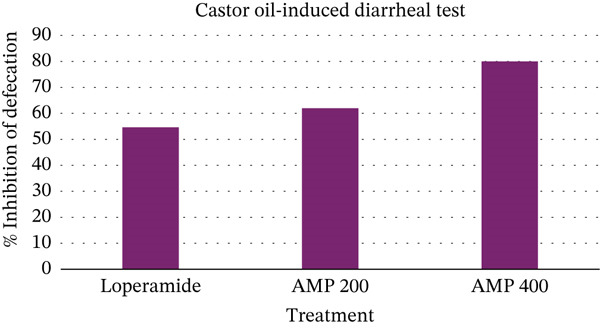
Antidiarrheal activity of acetone extract of *M. paniculatus* (AMP) on the castor oil‐induced diarrheal test.

An antidiarrheal study was carried out utilizing the castor oil–induced diarrhea model, a proven experimental framework to assess how well drugs control diarrhea (Table 5). In this investigation, the effects of the AMP extract were compared with those of a control group and the common medication loperamide. Interestingly, the antidiarrheal efficacy of the AMP extract was dose‐dependent. Diarrheal symptoms were significantly reduced by AMP at a higher dose of 400 mg/kg. The quantity of feces decreased dramatically to 2.5 ± 1.04, indicating a remarkable 80% suppression of defecation (Figure 3). These findings strongly support the efficacy of *M. paniculatus* extract as an antidiarrheal agent.

**Table 5:** Effects of AMP in the castor oil–induced diarrheal test.

**Group**	**No. of feces in 4 h**	**% inhibition of defecation**
Control	12.5 ± 1.56	—
Loperamide	5.67 ± 1.40∗	54.64
AMP 200	4.75 ± 1.03∗∗∗	62
AMP 400	2.5 ± 1.04∗∗∗	80

Data are expressed as mean ± SEM (n = 5). Statistical analysis was performed using one‐way ANOVA followed by Dunnett’s post‐hoc test.

Abbreviation: AMP, acetone extract of *M. paniculatus*.

∗*p* < 0.05, ∗∗*p* < 0.01, and ∗∗∗*p* < 0.001 indicate significant differences compared with the control group.

#### 3.5.2. Charcoal‐Induced Gastrointestinal Motility Test

The gastrointestinal motility test was conducted using the charcoal meal transit method to evaluate the antidiarrheal potential of the AMP. Mice treated with AMP at 200 mg/kg showed 35.56% inhibition of intestinal transit (28.67 ± 1.76 cm). In comparison, the 400 mg/kg dose exhibited a marked 64.03% inhibition, with charcoal movement reduced to 16 ± 0.57 cm—the shortest distance among all groups (Table 6). This significant, dose‐dependent reduction in gut motility indicates that AMP effectively slows intestinal transit, supporting its potential as an antidiarrheal agent.

**Table 6:** Effects of AMP on gastrointestinal motility tests.

**Group**	**Total length of intestine (cm)**	**Distance travelled by charcoal (cm)**	**Protection (%)**
Control	58.58 ± 0.32	44.49 ± 0.85	—
Loperamide	56.58 ± 0.94	16.80 ± 1.22 ∗∗∗	62
AMP 200	51.67 ± 0.88	28.67 ± 1.76 ∗∗∗	35.56
AMP 400	53 ± 1.15	16 ± 0.57 ∗∗∗	64.03

*Note:* Data are expressed as mean ± SEM (*n* = 5). Statistical analysis was performed using one‐way ANOVA followed by Dunnett′s post hoc test.

Abbreviation: AMP, acetone extract of *M. paniculatus*.

∗*p* < 0.05, ∗∗*p* < 0.01, and ∗∗∗*p* < 0.001 indicate significant differences compared with the control group.

### 3.6. In Silico Study

#### 3.6.1. ADME and Drug‐Likeness Analysis

A total of six phytochemicals identified from the AMP leaves were evaluated for their pharmacokinetic and toxicological profiles using in silico ADME analysis (Table 7). The evaluation focused on key parameters across the ADME spectrum: absorption (including human intestinal absorption and water solubility), distribution (volume of distribution and blood–brain barrier permeability), metabolism (CYP3A4 substrate status), and excretion (total clearance). Toxicity assessments included predictions for AMES mutagenicity and hepatotoxicity. Furthermore, drug‐likeness was assessed using Lipinski′s rule of five to evaluate compliance with key molecular properties associated with oral bioavailability. Predicted oral bioavailability was also determined to gauge the overall drug‐like potential of these compounds. The analyses were performed using computational tools such as admetSAR and ProTox 3.0, which provide reliable QSAR‐based predictions for diverse ADMET endpoints, supporting the prioritization of bioactive phytochemicals for further pharmacological development.

**Table 7:** ADME and drug‐likeness analysis of AMP compounds.

**Compound name**	**Lipinski Rules**				**L** **i** **p** **i** **n** **s** **k** **i** ^′^ **s** **v** **i** **o** **l** **a** **t** **i** **o** **n** ≤ 1	**Veber′s Rules**		**HIA**	**BS**	**BBB**
	**M** **W** (**g**/**m** **o** **l**) < 500	**H** **B** **A** < 5	**H** **B** **D** ≤ 5	**Log** **p** ≤ 5		**n** **R** **B** ≤ 10	**T** **P** **S** **A** ≤ 140			
3(2H)‐Benzofuranone	134.13	2	0	1.2617	0	0	26.30 Å^2^	1	0.55	0.9804
Tetradecanoic acid, 10,13‐dimethyl‐, methyl ester	270.45	2	0	5.3525	1	13	26.30 Å^2^	0.976	0.55	0.9791
Phytol	296.53	1	1	6.3641	1	13	20.23 Å^2^	0.9846	0.55	0.9375
Hexadecanoic acid 2‐hydroxy‐1‐(hydroxymethyl) ethyl ester	330.5	4	2	4.7117	0	18	66.76 Å^2^	0.9374	0.55	0.8227
6,7‐Dimethoxy‐2‐(4‐methoxyphenethyl)‐4h‐	340.37	5	0	3.604	0	6	57.90 Å^2^	0.9731	0.55	0.9138
Squalene	410.72	0	0	10.605	—	15	0.00 Å^2^	0.9895	—	0.9442

Abbreviations: BBB, blood–brain barrier; BS, bioavailability score; HBA, hydrogen bond acceptors; HBD, hydrogen bond donors; HIA, human intestinal absorption; n RB, number of rotatable bonds; TPSA, topological polar surface area.

#### 3.6.2. Toxicity Evaluation

The toxicity of the docked phytochemicals was evaluated using in silico tools, including ProTox 3.0 and admetSAR, by analyzing multiple toxicological parameters such as immunotoxicity, toxicity class, LD_50_ (median lethal dose), hepatotoxicity, cytotoxicity, carcinogenicity, and mutagenicity **(**Table 8 **)**. These computational models leverage QSAR‐based predictions and curated databases to assess potential adverse effects based on chemical structure. The results of this comprehensive toxicity assessment are summarized in Table 6, providing insights into the safety profiles of the compounds and supporting their potential for further pharmacological development.

**Table 8:** Toxicity evaluation of the AMP compounds.

**Compound name**	**Predicted LD**_**50**_ **(mg/kg)**	**Predicted toxicity class**	**AMES toxicity**	**Acute oral toxicity**	**Hepatotoxicity**	**Carcinogenicity**	**Mutagenicity**	**Immunotoxicity**	**Cytotoxicity**
3(2H)‐Benzofuranone	5400	6	+	III	−	+	−	−	−
Tetradecanoic acid, 10,13‐dimethyl‐, methyl ester	5000	5	−	III	−	−	−	−	−
Phytol	5000	5	−	III	−	−	−	−	−
Hexadecanoic acid 2‐hydroxy‐1‐(hydroxymethyl) ethyl ester	1600	4	−	IV	−	−	−	−	−
6,7‐Dimethoxy‐2‐(4‐methoxyphenethyl)‐4 h‐	1190	4	−	III	+	−	−	+	−
Squalene	5000	5	−	III	−	−	−	−	−

#### 3.6.3. Molecular Docking

**Figure 4:**
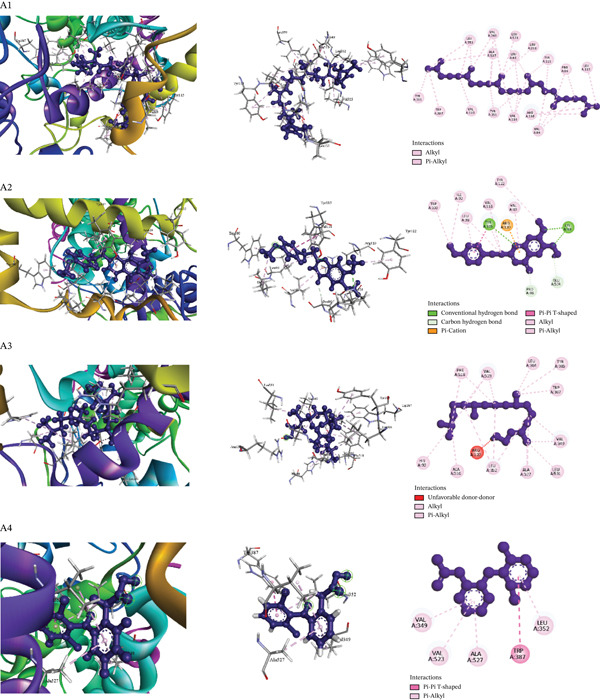
Molecular docking interaction of compounds against the cyclooxygenase‐2 (PDB: 6COX): A1. Squalene, A2. 6,7‐dimethoxy‐2‐(4‐methoxyphenethyl)‐4H‐, A3. Phytol and A4. Diclofenac (Standard).

**Figure 5:**
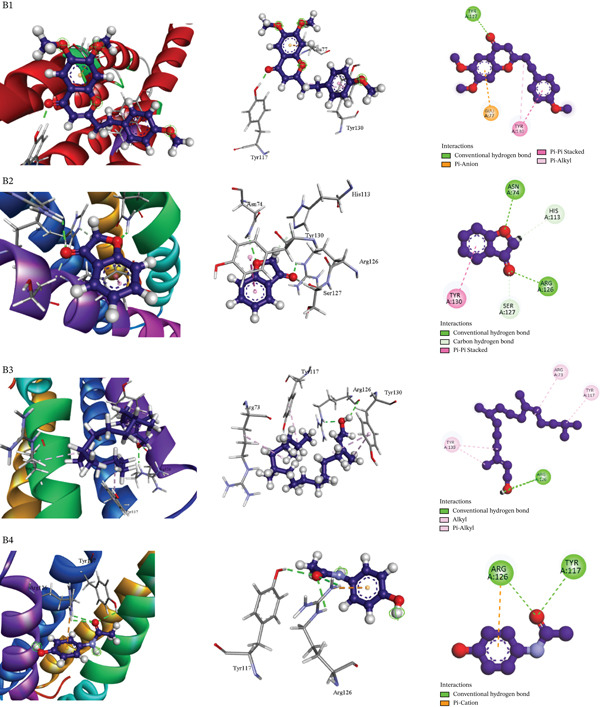
Molecular docking interaction of compounds against the microsomal prostaglandin E synthase‐1 (PDB: 4YK5): B1. 6,7‐dimethoxy‐2‐(4‐methoxyphenethyl)‐4H‐, B2. 3(2H)‐benzofuranone, B3. Phytol, and B4. Paracetamol (standard).

**Figure 6:**
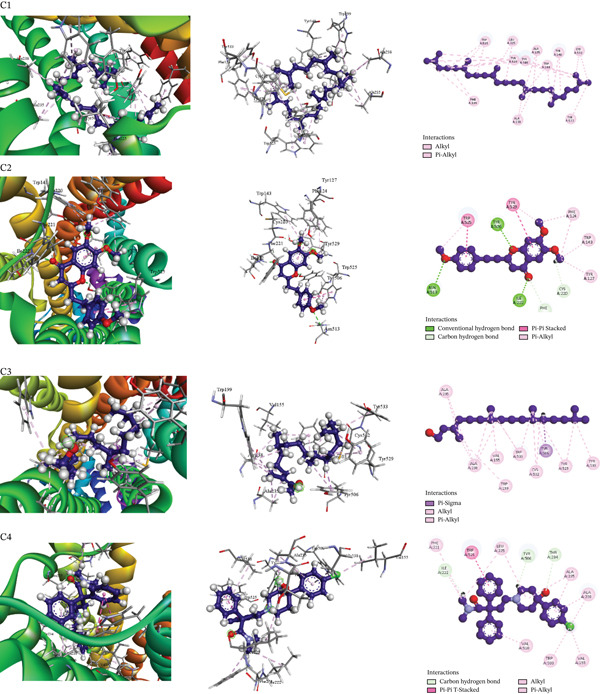
Molecular docking interaction of compounds against the M_3_ muscarinic acetylcholine receptor in complex with a selective antagonist (PDB: 5ZHP): C1. Squalene, C2. 6,7‐dimethoxy‐2‐(4‐methoxyphenethyl)‐4H‐, C3. Phytol and C4. Loperamide (standard).

Table 9 presents the docking scores of the extract′s phytochemicals against key receptors, including COX‐2 (PDB: 6COX), mPGES‐1 (PDB: 4YK5), and the M_3_ muscarinic acetylcholine receptor (PDB: 5ZHP). Table 10 and Figures 4, 5, and 6 illustrate the interactions of the best‐performing compound with the amino acid residues of these proteins. The figure provides a detailed visualization of the docking results, highlighting how the bioactive molecules bind to the active sites or other crucial regions of the target proteins, thereby offering insights into their possible mechanisms of action.

**Table 9:** Binding affinity and in silico binding interactions of AMP compounds.

**Sl. no**	**Compounds name**	**PubChem CID**	**Docking score (kcal/mol)**		
			**Antipyretic (4YK5)**	**Antidiarrheal (5ZHP)**	**Analgesic (6COX)**
1	3(2H)‐Benzofuranone	23556	−4.4	−6.1	−6.1
2	Tetradecanoic acid, 10,13‐dimethyl‐, methyl ester	554145	−3.8	−6.8	−6.6
3	Phytol	5280435	−4.2	−7.3	−7.2
4	Hexadecanoic acid 2‐hydroxy‐1‐(hydroxymethyl) ethyl ester	129853056	−3.9	−6.2	−6.8
5	6,7‐Dimethoxy‐2‐(4‐methoxyphenethyl)‐4 h‐	195226	−5.4	−8.5	−7.9
6	Squalene	638072	−4	−9.5	−8.1
7	Standard (paracetamol, loperamide, diclofenac)	—	−4	−8.8	−11.1

**Table 10:** Binding interactions of AMP compounds with selected receptors for analgesic (PDB: 6COX), antipyretic (PDB: 4YK5), and antidiarrheal activity (PDB: 5ZHP).

**Section number**	**Receptor**	**Compound name**	**Binding affinity (kcal/mol)**	**Bond type**	**Amino acids**
1	6COX	Squalene	−8.1	Alkyl	PRO86(2), VAL89(3), VAL116(3), VAL349(3), VAL523, ALA527(2), LEU93, ARG120(2), LEU123, LEU359, LEU531, LEU352(3)
				Pi–alkyl	TYR115(2), TYR355(2), TYR385, TRP387
		6,7‐Dimethoxy‐2‐(4‐methoxyphenethyl)‐4 h‐	−7.9	Conventional hydrogen bond	LYS83(2), TYR115)
				Carbon hydrogen bond	PRO86, GLU524
				Pi–cation	ARG120
				Pi–sigma	LEU93
				Pi–pi T‐shaped	TYR115
				Alkyl	VAL116, LEU93, ILE92
				Pi–alkyl	TRP100, TYR115, TYR122, VAL89
		Phytol	−7.2	Alkyl	VAL349(2), ALA516, VAL523(3), ALA527(2), LEU384, LEU352(3), LEU531
				Pi–alkyl	HIS90, TYR385, TRP387, PHE518(2)
		Diclofenac (Standard)	−8.4	Pi–pi T‐shaped	TRP387
				Pi–alkyl	VAL349, VAL523, ALA527, LEU352

2	4YK5	6,7‐Dimethoxy‐2‐(4‐methoxyphenethyl)‐4 h‐	−5.4	Conventional hydrogen bond	TYR117
				Pi–anion	GLU77
				Pi–pi stacked	TYR130
				Pi–alkyl	TYR130
		3(2H)‐Benzofuranone	−4.4	Conventional hydrogen bond	ASN74
				Conventional hydrogen bond	ARG126
				Carbon hydrogen bond	SER127
				Carbon hydrogen bond	HIS113
				Pi–pi stacked	TYR130
		Phytol	−4.2	Conventional hydrogen bond	ARG126 (2)
				Alkyl	ARG73
				Pi–alkyl	TYR117
				Pi–alkyl	TYR130 (2)
		Paracetamol (standard)	−4	Conventional hydrogen bond	TYR117
				Conventional hydrogen bond	ARG126 (2)
				Pi–cation; Pi‐donor hydrogen bond	ARG126

3	5ZHP	Squalene	−9.5	Alkyl	ALA235(2), ALA238 (2), LEU225 (2), CYS532 (2)
				Pi–alkyl	PHE124 (2), TYR148 (2), TRP199 (3), TYR506 (2), TRP525 (5), TYR529 (5), TYR533
		6,7‐Dimethoxy‐2‐(4‐methoxyphenethyl)‐4 h‐	−8.5	Conventional hydrogen bond	ILE222, TYR506, ASN513,
				Carbon hydrogen bond	PHE221, CYS220
				Pi–pi stacked	TRP525 (2), TYR529
				Pi–alkyl	PHE124 (2), TYR127, TRP143, TRP525 (2), TYR529
		Phytol	−7.3	Pi–sigma	TYR506
				Alkyl	ALA235 (2), ALA238 (4), CYS532 (2), VAL155
				Pi–alkyl	TRP199, TRP503 (4), TYR506, TYR529 (2), TYR533
		Loperamide (standard)	−8.8	Carbon hydrogen bond	THR234, TYR506, ILE222
				Pi–pi stacked	TRP525 (2)
				Alkyl	LEU225, ALA235, ALA238, ILE222, LEU225, VAL155
				Pi–alkyl	PHE221, TRP503, ALA235, ALA238, VAL510

##### 3.6.3.1. Docking Analysis for Analgesic Activity.

The interaction of each compound with the COX‐2 enzyme (PDB ID: 6COX) was evaluated through molecular docking to assess their potential analgesic activity. Among the tested compounds, squalene exhibited the highest binding affinity with a docking score of −8.1 kcal/mol, performing closely to the reference drug diclofenac (−8.4 kcal/mol). The following most active compounds were 6,7‐dimethoxy‐2‐(4‐methoxyphenethyl)‐4H‐chromen‐4‐one (−7.9 kcal/mol) and phytol (−7.2 kcal/mol). A detailed analysis of the docking pose revealed that squalene formed 29 strong intermolecular interactions—primarily hydrophobic and van der Waals contacts—with key amino acid residues in the active site of COX‐2, all at short distances. This extensive network of interactions indicates high binding stability and affinity for the target. The binding mode of squalene closely resembles that of diclofenac, suggesting a similar mechanism of enzyme inhibition. Thus, it may serve as a promising natural lead compound for the development of an analgesic agent. The results are summarized in Table 10 (Section 1) and visualized in Figure 4.

##### 3.6.3.2. Docking Analysis for Antipyretic Activity.

The antipyretic potential of selected bioactive compounds from *M. paniculata*s leaves was evaluated through molecular docking analysis against mPGES‐1 (PDB ID: 4YK5), a key enzyme involved in fever mediation. All tested compounds exhibited notable binding affinity to the target receptor, which correlates with their potential antipyretic activity. Of particular interest, 6,7‐dimethoxy‐2‐(4‐methoxyphenethyl)‐4H‐chromen‐4‐one demonstrated the highest binding affinity at −5.4 kcal/mol, outperforming the reference drug paracetamol (−4.0 kcal/mol). Detailed docking analysis revealed that this compound formed four strong interactions with critical amino acid residues in the active site of mPGES‐1, including Tyr117, Glu77, and Tyr130 (2) [Table 10 (Section 2) and Figure 5]. These interactions suggest stable and specific binding within the catalytic or allosteric site of the enzyme, thereby potentially inhibiting its activity. The strong binding affinity and favorable interaction profile highlight the compound’s promise as a potent natural antipyretic agent, warranting further experimental validation.

##### 3.6.3.3. Docking Analysis for Antidiarrheal Activity.

All tested compounds demonstrated potential antidiarrheal activity through successful molecular docking with the M_3_ muscarinic acetylcholine receptor (PDB: 5ZHP), a key target involved in regulating gastrointestinal motility and secretion. Notably, squalene exhibited the highest binding affinity of −9.5 kcal/mol, surpassing that of the reference antidiarrheal drug loperamide (−8.8 kcal/mol), suggesting stronger interaction with the receptor and enhanced inhibitory potential. Furthermore, the most active compounds also showed significant affinity for the human serotonin transporter (SERT) active site, forming strong intermolecular interactions—such as hydrogen bonds and hydrophobic contacts—with critical amino acid residues at short distances, which may contribute to modulation of gut motility and secretion. As supported by the data in Table 10 (Section 3) and Figure 6, these dual‐target interactions indicate that the compounds, particularly squalene, possess promising pharmacological potential as antidiarrheal agents. The results highlight their ability to act via both cholinergic and serotonergic pathways, warranting further experimental validation.

### 3.7. PASS Prediction

The compounds present in AMP were evaluated using PASS prediction analysis to assess their potential analgesic, antipyretic, and antidiarrheal activities, and the outcomes are summarized in Table 11.

**Table 11:** PASS prediction of biologically active AMP compounds.

**Compounds**	**Biological activity**					
	**Analgesic**		**Antipyretic**		**Antidiarrheal**	
	**P** **a**	**P** **i**	**P** **a**	**P** **i**	**P** **a**	**P** **i**
3(2H)‐Benzofuranone	0.411	0.101	0.245	0.075	0.174	0.145
Tetradecanoic acid, 10,13‐dimethyl‐, methyl ester	0.465	0.059	0.219	0.097	0.289	0.092
Phytol	0.300	0.182	0.206	0.074	0.506	0.027
Hexadecanoic acid 2‐hydroxy‐1‐(hydroxymethyl) ethyl ester	0.391	0.116	0.526	0.011	0.531	0.023
6,7‐Dimethoxy‐2‐(4‐methoxyphenethyl)‐4h‐	0.271	0.206	0.204	0.110	0.367	0.061
Squalene	0.474	0.053	0.245	0.042	0.453	0.038

## 4. Discussion

For many years, herbal therapy has offered safe, all‐natural remedies for various complex diseases. Owing to their structural diversity and broad pharmacological activities, natural products have become a significant focus of drug discovery, particularly because they often exhibit lower toxicity compared with synthetic compounds [55]. The unique compounds and wide range of therapeutic effects of medicinal plants are making them increasingly valuable in drug research. Many of the bioactive compounds found in these plants have the potential to improve human health and treat various diseases [56]. Although many medications have been developed to treat helminthiasis, cancer, depression, anxiety, and insomnia, it is still unclear how to completely eradicate disease symptoms without causing harm. The poor pharmacokinetics and adverse effects of these medicines restrict their therapeutic usage. As a result, concerns about the efficacy, safety, duration of action, and side effects of new pharmaceuticals have grown in importance, and there is a growing need for novel therapies [57]. The ethnomedicinal plant *M. paniculatus* was selected to examine its numerous pharmacological characteristics. Previous research on the constituents of *M. paniculatus* has identified a wide range of chemicals, including cardenolides, triterpenoids, steroids, flavonoids, and unsaturated fatty acids [58]. Antioxidant, antibacterial, antifungal, and anticancer activities were also discovered in the plant extract [18]. In our research, we have demonstrated the various thrombolytic, antibacterial, and neuropharmacological properties of this plant.

Through a variety of catabolism and anabolism processes, the human body generates thousands of chemical reactions. Reactive oxygen and nitrogen species (RONS) and inflammatory mediators cause many reactions that promote pain, inflammation, and oxidative stress [59]. Receptors such as TLRs (toll‐like receptors) and NLRs (NOD‐like receptors) trigger the rush of defense cells, such as leukocytes, to the site of injury when there is inflammation, such as from microbial infection or tissue damage [60]. Eicosanoids, cytokines, chemokines, and vasoactive amines are among the many inflammatory mediators that can cause inflammation [61]. In mice using the acetic acid–induced writhing method, a greater peripheral analgesic effect is correlated with both a decrease in the number of writhes and an increase in the percentage of writhing inhibition. Similar to standard, AMP demonstrated a significant (*p* < 0.001) suppression of writhing. Notably, a significantly greater impact was achieved by increasing the dose to 400 mg/kg, which marginally outperformed the conventional medication. Writhes were reduced to 16.33 ± 0.88, and the inhibition was 53.77%. AMP′s ability to suppress acetic acid–induced writhing may stem from the delayed activity of prostaglandins. The central and peripheral analgesic effects of the examined extracts were also evaluated using the formalin‐induced hind paw licking test. Two distinct periods of analgesia are correlated with this test. Two hundred milligrams per kilogram of AMP dramatically decreased paw licking in the early phase (56.18% inhibition) and the late phase (69.4% inhibition), outperforming morphine in the latter phase, suggesting great anti‐inflammatory or central analgesic potential. Furthermore, the tail immersion technique is also an effective way to screen for centrally acting analgesic effects [62]. Reaction times improved significantly (*p* < 0.05) from 30 to 120 min for all AMP groups, but none of them exhibited an early analgesic effect.

The inhibition of prostaglandin production, which happens when paracetamol inhibits cyclooxygenase and hence prevents prostaglandin synthesis, is one possible explanation for the action of antipyretic medications [63]. The suppression of many pyrexia‐causing mediators is the cause of the antipyretic action [64]. Rectal temperature increased significantly following an 18‐h subcutaneous dose of brewer′s yeast suspension. After 3 h, AMP at 400 mg/kg demonstrated the most significant reduction in pyrexia, with an effect similar to that of paracetamol at 100 mg/kg, the conventional treatment. The study provides evidence that AMP exhibits antipyretic properties. It merits investigation to determine the likely mechanism of action, which might be either accelerated drug dissipation at the temperature or interference with heat production resulting in pyrexia. The formulation′s herbal constituents contain secondary metabolites, which may be the cause of the antipyretic action. Natural sources of COX‐2 inhibitors are assumed to have fewer harmful effects than synthetic medications, which can harm many bodily organs.

Gastrological motility and the castor oil–induced diarrhea test, two popular techniques for assessing antidiarrheal effects and gut motility regulation, were employed to evaluate the antidiarrheal activity of AMP. Intestinal motility and fluid secretion are induced in mice to cause diarrhea in the castor oil–induced diarrhea test [65]. The antidiarrheal effects of AMP 400 mg/kg were similar to those of loperamide, as it significantly inhibited diarrhea and reduced feces. A notable 80% inhibition of defecation was indicated by the sharp drop in fecal volume to 2.5 ± 1.04. These results provide compelling evidence for the effectiveness of the extract from AMP as an antidiarrheal medication. Additionally, the gastrointestinal motility test is used to determine the impact of AMP on the duration of time a charcoal meal travels through the digestive tract [65]. The experimental groups were given 200 mg/kg and 400 mg/kg of AMP. Intestinal movement was inhibited by 64.03% and charcoal travelled only 16 ± 0.57 cm at the 400 mg/kg dose, demonstrating significant antidiarrheal action. To confirm the antidiarrheal efficacy of the root extract of AMP, the possible mode of action was studied using enteropooling and antimotility tests in the intestine. This finding implied that one of the mechanisms underlying the root extract′s antidiarrheal effects might be its antisecretory effect. The study suggests that the plant has the potential to be utilized in the development of novel medicines for treating diarrhea.

Molecular docking studies, which are essential for rational medication development, were employed in this study to support the biological testing investigations. To elicit the exact pharmacological action, this technique focuses on understanding how a ligand, a small molecule, binds chemically to the binding pocket of a receptor, which is typically a protein. A computer technique called protein–ligand docking is used to forecast the exact placement and conformation of a ligand within the binding site of a known protein structure. This method provides essential insights into the potential mechanisms of action of the compounds under study. It aids in determining the molecular interactions that give rise to the observed biological effects [66]. The compounds extracted from the acetonic extract of *M. paniculatus* demonstrated strong interactions with key target proteins, including COX‐2 (PDB: 6COX), muscarinic acetylcholine receptor M_3_ (PDB: 5ZHP), and mPGES‐1 (PDB: 4YK5), in the assessment of analgesic, antidiarrheal, and antipyretic activities. Interaction scores ranging from −3.8 to −11.1 kcal/mol suggest that these compounds hold significant potential for chemical optimisation of leads. Furthermore, the PASS prediction data presented in Table 11 corroborated these findings, reinforcing the therapeutic relevance of the identified compounds.

## 5. Conclusion

This study demonstrates that the *M. paniculatus* (AMP) possesses significant analgesic, antipyretic, and antidiarrheal activities. Both in vivo analyses revealed that AMP, particularly at higher doses, effectively reduced pain, fever, and diarrhea in experimental models, with molecular docking results further supporting its pharmacological potential. These outcomes validate the traditional use of *M. paniculatus* in treating a wide range of complaints and suggest that AMP could be used as a promising source for the development of novel therapeutic agents. Further studies, including identifying the active compounds, exploring the mechanisms in detail, and conducting clinical assessments, are recommended to validate its safety and effectiveness fully.

## Author Contributions

**M.J.I.M.:** planning, designing, investigation, data curation, data analysis, software, and writing—original draft; **M.A.A.:** planning, designing, data curation, data analysis, software, and writing—original draft; **S.T.T.:** planning, designing, data curation, data analysis, and writing—original draft; **M.I.H.:** data curation and data analysis; **S.C.D.:** data curation and data analysis; **T.A.M.:** writing—original draft, and conceptualization (equal), **D.D.:** writing—original draft and conceptualization (equal); **S.A.**: writing—original draft and conceptualization (equal); **S.M.M.H.:** conceptualization (lead), planning, designing, investigation, data curation, data analysis, writing—original draft, and writing—review and editing (lead).

## Funding

No funding was received for this manuscript.

## Disclosure

The authors thoroughly revised the content to ensure the accuracy and integrity of the final published work.

## Conflicts of Interest

The authors declare no conflicts of interest.

## Supporting information


**Supporting Information** Additional supporting information can be found online in the Supporting Information section. Figure S1: This figure contains the GC‐MS chromatogram of the acetone extract of *M. paniculatus.* Table S1: This table contains compounds identified by GC‐MS analysis in the *M. paniculatus.*

## Data Availability

The raw data supporting the findings of this study will be available from the authors.
