# Supplementary material for: Pharmacological and Computational Insights Into the Analgesic, Antipyretic, and Antidiarrheal Potential of Mallotus paniculatus Acetone Extract
Source: ScientificWorldJournal. 2026 Mar 3;2026:9813151. doi: 10.1155/tswj/9813151 (PMC12957538; doi:10.1155/tswj/9813151)
Supplement: Supplementary file 1 — Supporting Information Additional supporting information can be found online in the Supporting Information section. Figure S1: This figure contains the GC‐MS chromatogram of the acetone extract of M. paniculatus. Table S1: This table contains compounds identified by GC‐MS analysis in the M. paniculatus. [file TSWJ-2026-9813151-s001.docx]

**Supplementary Figure 1.** GC-MS chromatogram of the acetone extract of *Mallotus paniculatus*.

**Supplementary Table 1.** GC-MS compounds of the acetone extract of *Mallotus paniculatus*.

| **SL.NO.** | **RT** | **Compound Name** | **M.W.** | **Formula** | **Area** | **Sum of area** | **% of area** |
| --- | --- | --- | --- | --- | --- | --- | --- |
| 1 | 4.59 | 3(2H)-Benzofuranone | 134.13 | C_8_H_6_O_2_ | 3078632 | 39161921 | 7.86129 |
| 2 | 11.41 | Tetradecanoic acid, 10,13-dimethyl-,  methyl ester | 270.5 | C_17_H_34_O_2_ | 545365 | 39161921 | 1.39259 |
| 3 | 13.21 | Phytol | 296.5 | C_20_H_40_O | 18810434 | 39161921 | 48.03246 |
| 4 | 16.77 | Hexadecanoic acid 2-hydroxy-1-  (hydroxymethyl) ethyl ester | 330.5 | C_19_H_38_O_4_ | 3936300 | 39161921 | 10.05135 |
| 5 | 18.97 | 6,7-dimethoxy-2-(4-  methoxyphenethyl)-4H- | 340.4 | C_20_H_20_O_5_ | 1918080 | 39161921 | 4.897819 |
| 6 | 19.43 | Squalene | 410.7 | C_30_H_50_ | 10873110 | 39161921 | 27.7645 |
